# Prognostic Role of Blood Markers in Primary Central Nervous System Lymphoma Patients Treated With High-Dose Methotrexate-Based Therapy

**DOI:** 10.3389/fonc.2021.639644

**Published:** 2021-04-29

**Authors:** Qian Luo, Chunli Yang, Chunxi Fu, Wanchun Wu, Yi Wei, Liqun Zou

**Affiliations:** ^1^Department of Medical Oncology, Cancer Center, West China Hospital, Sichuan University, Chengdu, China; ^2^Department of Central Medical Transportation, West China Hospital, Sichuan University, Chengdu, China

**Keywords:** primary central nervous system lymphoma, prognosis, blood marker, C-index, MSKCC

## Abstract

**Purpose:** Primary central nervous system lymphoma (PCNSL) is a rare type of extra-nodal non-Hodgkin lymphoma, but the prognostic value of blood parameters indicating systemic inflammation and nutritional status remains unknown. We aim to explore the prognostic role of blood parameters in PCNSL.

**Methods:** All PCNSL patients diagnosed at West China Hospital between February 2011 and February 2020 were retrospectively screened. For patients who were initially treated with high-dose methotrexate (HD-MTX)-based therapy, clinical data were collected. Survival analyses were performed using the Kaplan–Meier method and multivariable Cox proportional regression. The accuracies of different multivariate models were assessed by Harrell's *C* statistical analysis (*C*-index).

**Results:** Sixty patients were included. Median overall survival (OS) was 4.8 ± 3.7 years, and median progression-free survival (PFS) was 1.9 ± 1.3 years. In the multivariate analysis, hemoglobin (Hb) (HR 3.940, *p* = 0.013), neutrophil–lymphocyte ratio (NLR) (HR 10.548, *p* = 0.034), and total bilirubin (TBIL) (HR 3.429, *p* = 0.004) had independent prognostic values for PFS, while lymphocyte–monocyte ratio (LMR) (HR 6.195, *p* = 0.039), systemic immune-inflammation index (SII) (HR 5.144, *p* = 0.012), and TBIL (HR 3.892, *p* = 0.009) were independently related to OS. The *C*-index of the Memorial Sloan-Kettering Cancer Center (MSKCC) score increased from 0.57 to 0.72 when SII and TBIL were combined.

**Conclusions:** Our study indicated that pretreatment Hb, NLR, SII, LMR, and TBIL were convenient prognostic factors in PCNSL. Adding SII and TBIL to the MSKCC score can better predict the survival of PCNSL based on HD-MTX regimens.

## Introduction

Primary central nervous system lymphoma (PCNSL) is a rare type of extra-nodal non-Hodgkin lymphoma, accounting for 3% of brain tumors (1). It usually occurs in the brain parenchyma, leptomeninges, spinal cord, or eyes and is typically confined to the central nervous system (CNS). More than 90% of PCNSLs belong to the diffuse large B-cell lymphoma (DLBCL), and non-germinal center B cell (non-GCB) is the most common pathology phenotype (2–4). The initial standard high-dose methotrexate (HD-MTX) regimen produced a high objective response rate (ORR) of around 50–90% (5, 6). Nevertheless, about one third of patients presented no response to the standard therapy, and half of the responders inevitably relapsed (7). Thus, identifying patients of inferior outcomes with standard therapy is an unmet need in clinical practice.

Several prognostic scores have been proposed for stratifying the risk groups and estimating the survival time of different groups in PCNSL. In DLBCL, the International Prognostic Index (IPI) is the frequently used model for prognosis, for which administration has been limited in PCNSL due to the two unfit IPI variables (stage of disease and number of extra-nodal sites) (8). Therefore, the International Extra-nodal Lymphoma Study Group (IELSG) score and the Memorial Sloan-Kettering Cancer Center (MSKCC) score were proposed for the risk stratification in PCNSL. Five binary clinical variables, namely, age, Eastern Cooperative Oncology Group performance status (ECOG-PS), lactate dehydrogenase (LDH), cerebrospinal fluid (CSF) protein concentration, and deep brain involvement, were used to distinguish three risk groups in IELSG (8). As CSF protein data are frequently lacking in clinical practice, it is difficult to perform the IELSG score for prognosis and verify its predictive power (3, 9, 10). In the MSKCC score, Karnofsky performance status (KPS) ≥ 70 and age ≥ 50 years were used as two prognostic factors dividing patients into three risk groups (11). This score is simpler while still controversial in several recent studies (9, 12, 13). Furthermore, these prognostic models need to be confirmed in the modern combination chemotherapy regimens based on HD-MTX and should be optimized to better distinguish risk groups.

New prognostic factors ought to be cheap for patients, be convenient for clinicians, and improve the risk assessment in PCNSL with standard treatment. Among these commonly available clinical biomarkers at baseline, complete blood cell counts (CBCs) and liver serum markers have been proven to be prognostic factors in several cancers including lymphoma (14–18). In patients with PCNSL, the prognostic value of peripheral blood parameters indicating systemic inflammation and nutritional status remains undefined.

This study aims to assess the prognostic role of these baseline blood parameters in PCNSL and improve the previously reported prognostic models.

## Method

We retrospectively reviewed the clinical and laboratory data of patients newly diagnosed with PCNSL of DLBCL type who were treated at West China Hospital between February 2011 and February 2020. The inclusion criteria were the following: (a) pathologically diagnosed with DLBCL confined to the brain, spinal cord, cranial nerves, eyes, and meninges according to the 2008 or 2016 World Health Organization classification (19, 20); (b) initially received at least one cycle of HD-MTX-based induction therapy; (c) had available baseline clinical and laboratory data prior to initial chemotherapy. The exclusion criteria were (a) HIV-positive status, history of organ transplantation, and previous diagnosis of systemic DLBCL or other CNS cancers and (b) evidence of systemic DLBCL from computed tomography (CT), magnetic resonance imaging (MRI), or positron emission tomography CT (PET-CT) of the chest, abdomen, pelvis, and bone marrow aspirate and biopsy. The follow-up information was obtained from medical records or telephone interviews. Response assessment was routinely performed every two cycles of HD-MTX-based therapy according to international guidelines (21). This study was approved by the Ethics Administration Office of West China Hospital.

Baseline clinical characteristics, including gender, age, ECOG-PS, location and number of lesions, level of LDH, CSF protein, and the process of treatment and outcomes, were retrospectively collected. All included laboratory parameters were assessed prior to the start of chemotherapy and glucocorticoid treatment usually within 7 days, containing absolute neutrophil counts, absolute lymphocyte counts (ALCs), absolute platelet counts, absolute monocyte counts, hemoglobin (Hb), serum albumin, and total bilirubin (TBIL). For patients who received glucocorticoid treatment before diagnosis, blood tests were obtained after at least 7 days of glucocorticoid-free treatment. The prognostic nutritional index (PNI) is an indicator of nutritional status and systemic inflammation, calculated as serum albumin (g/L) + 5 × ALC (× 10^9^/L) (22). Systemic immune-inflammation index (SII) is a systemic inflammation response indicator calculated as platelet counts × neutrophil counts/lymphocyte counts (16).

Progression-free survival (PFS) was defined as the time from diagnosis to disease progression, relapse, death from any cause, or censored alive. Overall survival (OS) was defined as the time from diagnosis to death from any cause or censored alive. Cutoff values were determined using the MaxStat package, which iteratively tests all possible cutoff points in order to achieve the maximum log-rank statistic (23).

Pearson chi-square test, Fisher's exact test, Student's *t*-test, and Mann–Whitney analyses were used for univariate analysis, where appropriate. PFS and OS were estimated by Kaplan–Meier curve analysis, and the differences between groups were compared by log-rank test. Cox regression models were used for univariate and multivariate analyses, and only statistically significant factors were included in the multivariate Cox analysis. Different multivariate Cox models were compared by Harrell's *C* statistical analysis (*C*-index) (24). A *p*-value < 0.05 was considered statistically significant, and all tests were two-sided. All statistical analyses were performed by Statistical Package for the Social Sciences (SPSS version 21.0) and R software (version 4.0.2).

## Results

### Patient Characteristics

A total of 60 patients diagnosed with PCNSL, initially treated with HD-MTX-based induction therapy at West China Hospital between February 2011 and February 2020, were included in the final analysis. The clinical characteristics and treatments were detailed in Table 1. The median age was 57 years (range 18–79) at the time of diagnosis with 43.3% of patients older than 60 years. There was a mild predominance of male (55%), and 20 patients had good ECOG-PS of 0–1 before chemotherapy. Only one patient had right-eye involvement except the brain. Twenty-five patients (41.7%) presented with a single brain lesion, and 28 patients (46.7%) had involvements of deep-brain structures. Forty-four (73.3%) patients had measurable disease before chemotherapy. Twelve patients (20%) had an elevated value of LDH. Elevated CSF protein was founded in 17 (65.4%) of 26 patients through intrathecal diagnostic evaluation before chemotherapy. The MSKCC score divided patients into three risk groups: 12 patients (20%) in class 1, 22 patients (36.7%) in class 2, and 26 patients (43.3%) in class 3.

**Table 1:** Baseline characteristics of patients with PCNSL.

**Characteristics**	**Overall (*N* = 60)**
**Age (years old)**	
Median (range)	57 (18–79)
≥60	26 (43.3%)
<60	34 (56.7%)
**Sex**	
Male	33 (55.0%)
Female	27 (45.0%)
**ECOG-PS**	
0–1	20 (33.3%)
>1	40 (66.7%)
**Extra-CNS involvement**	
Absent	59 (98.3%)
Present	1 (1.7%)
**Diagnostic method**	
Surgical excision	47 (78.3%)
Stereotactic biopsy	12 (20.0%)
CSF	1 (1.7%)
**Number of lesions**	
Multiple	25 (41.7%)
Single	35 (58.3%)
**Involvement of deep brain structures**	
Yes	28 (46.7%)
No	32 (53.3%)
**Measurable disease before chemotherapy**	
Yes	44 (73.3%)
No	16 (26.7%)
**Corticosteroids before diagnosis**	17 (28.3%)
**LDH**	
>ULN	12 (20.0%)
≤ ULN	48 (80.0%)
High CSF protein (>45 mg/dL)	17/26 (65.4%)
**MSKCC**
Age < 50 years	12 (20.0%)
Age ≥ 50 years and KPS ≥ 70	22 (36.7%)
Age ≥ 50 years and KPS < 70	26 (43.3%)
**Induction therapy**	
HD-MTX	18 (30.0%)
MA	15 (25.0%)
MVD	14 (23.3%)
Others	13 (21.7%)
+Rituximab	45 (75.0%)
**Initial response to induction therapy**	
CR	21 (35.0%)
CRu	7 (11.7%)
PR	11 (18.3%)
SD	3 (5.0%)
PD	10 (16.7%)
Not available	8 (13.3%)
**Consolidation treatment**	
WBRT	17/39 (43.6%)
ASCT	2/39 (5.1%)
Others	7/39 (17.9%)
No	13/39 (33.3%)

*ECOG-PS, Eastern Cooperative Oncology Group performance status; CNS, central nervous system; LDH, lactate dehydrogenase; ULN, upper limit of normal; CSF, cerebrospinal fluid; MSKCC, Memorial Sloan Kettering Cancer Center; KPS, Karnofsky Performance Status; HD-MTX, high dose methotrexate; MA, high dose cytarabine and HD-MTX; MVD, HD-MTX, vincristine, dexamethasone; CR, complete response; CRu, unconfirmed complete response; PR, partial response; SD, stable disease; PD, progressive disease; WBRT, whole brain radiation therapy; ASCT, autologous stem cell transplantation*.

All patients received at least one cycle of HD-MTX-based induction therapy, including (a) HD-MTX; (b) HD-MTX and high-dose cytarabine (MA); (c) HD-MTX, vincristine, and dexamethasone (MVD); and others. Forty-five patients received rituximab combined treatments. Twenty-three (38.3%) patients had grade 3–4 treatment-related hematological adverse events. Serious pneumonia occurred in two (3.3%) patients. In our cohort, the ORR was 65%, and the complete response (CR) rate (CR and CRu) was 47% after induction therapy. Of the 39 patients who achieved treatment response, 18 (46.2%) patients received whole-brain radiation therapy (WBRT) as consolidation treatment. Autologous stem cell transplantation (ASCT) was performed in two (5.1%) patients. Seven patients received other consolidation treatments like ibrutinib, lenalidomide, and/or temozolomide. Treatment of relapsed and refractory PCNSL patients included WBRT, other immunochemotherapies, temozolomide, and best supported care. The median follow-up time was 34 months (range 0–83). Median OS was 4.8 ± 3.7 years, and median PFS was 1.9 ± 1.3 years. The 2-year OS rate was 59.55%, and the 2-year PFS rate was 46.1%. During the observation period, 27 deaths occurred. Five patients died of pneumonia. Two deaths were related to postoperative complications. One patient died of accident trauma. Thirteen deaths were due to disease progression, and others died of unknown causes.

### Survival Analysis

**Figure 1:**
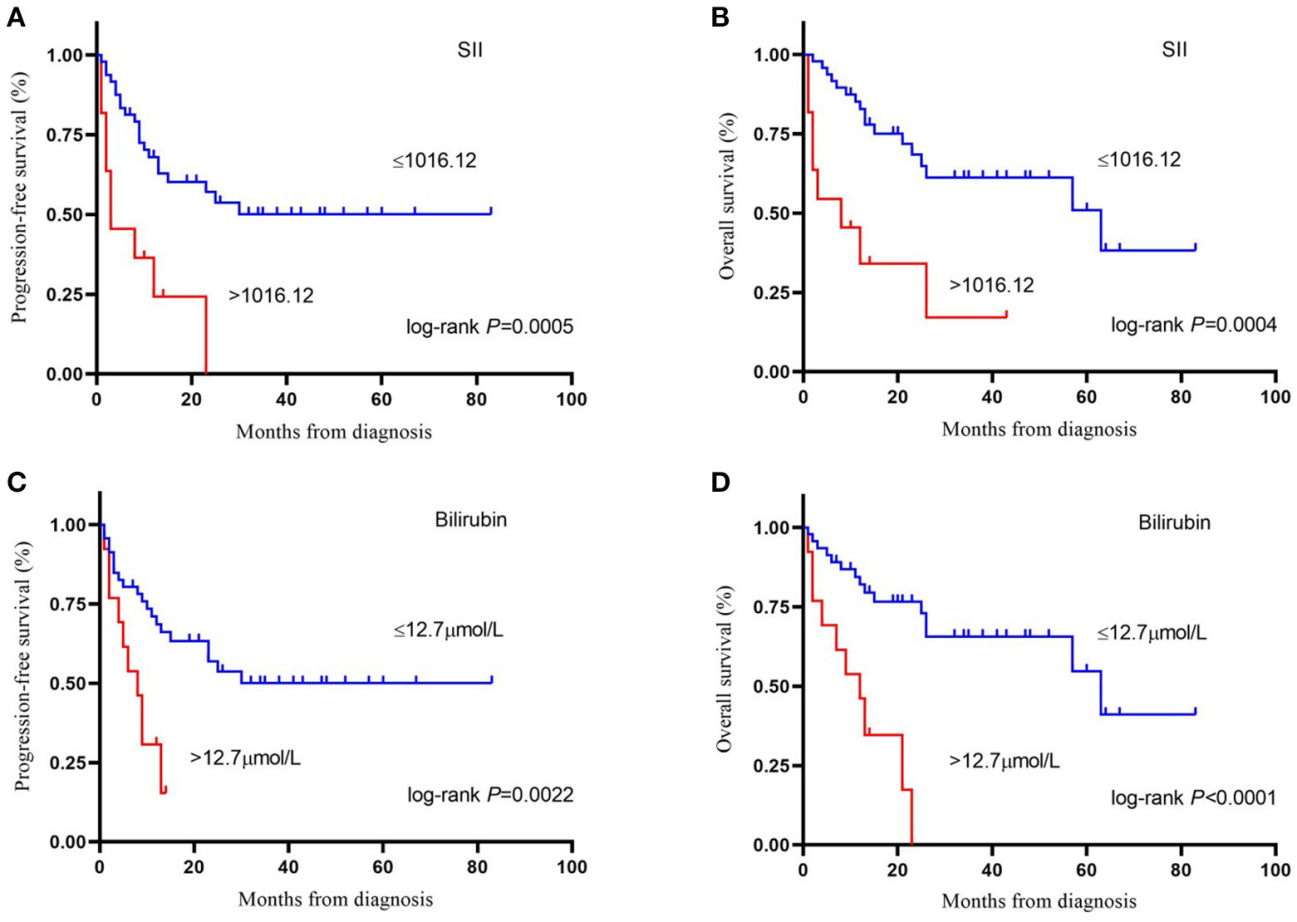
Kaplan-Meier survival curve of progression–free survival **(A)** and overall survival **(B)** by systemic immune-inflammation index (cut-off = 1,016.12). Kaplan-Meier survival curve of progression–free survival **(C)** and overall survival **(D)** by bilirubin (cut-off = 12.7 μmol/L). SII, systemic immune-inflammation index.

Cutoff values of several serum markers have been separately determined by the MaxStat package for their influence on PFS and OS (Table 2). In the univariate analysis (Table 3), the following factors significantly associated with worse PFS: Hb (g/dl) ≤ 11.1 (*p* = 0.050), neutrophil count (10^9^/L) > 3.28 (*p* = 0.013), platelet counts (10^9^/L) > 234 (*p* = 0.015), neutrophil–lymphocyte ratio (NLR) > 1.79 (*p* = 0.034), platelet–lymphocyte ratio (PLR) > 243.75 (*p* = 0.017), SII > 1,016.12 (*p* = 0.000), TBIL (mg/dl) > 0.74 (*p* = 0.007), and PNI > 47.65 (*p* = 0.017). The Kaplan–Meier survival curves are shown in Figures 1A,C. Three factors were significant independent predictors in multivariate analysis for PFS (Table 3): Hb (g/L) ≤ 111 (*p* = 0.013), NLR > 1.79 (*p* = 0.034), and TBIL (mg/dl) > 0.74 (*p* = 0.004).

**Table 2:** Cut-off values for PFS and OS.

**Variable**	**Reference value**	**Cut-off value (PFS)**	**Overall (*N* = 60)**	**Cut-off value (OS)**	**Overall (*N* = 60)**
Hemoglobin (g/dL)	13.0~17.5	>11.1	51 (85.0%)	>15.0	6 (10.0%)
		≤11.1	9 (15.0%)	≤15.0	54 (90.0%)
Neutrophil counts (10^9^/L)	1.8~6.3	≤3.28	17 (28.3%)	≤3.28	17 (28.3%)
		>3.28	43 (71.7%)	>3.28	43 (71.7%)
Lymphocyte counts (10^9^/L)	1.1~3.2	≤2.56	54 (90.0%)	>1.68	32 (53.3%)
		>2.56	6 (10.0%)	≤1.68	28 (46.7%)
Monocyte counts (10^9^/L)	0.1~0.6	≤0.19	6 (10.0%)	≤0.19	6 (10.0%)
		>0.19	54 (90.0%)	>0.19	54 (90.0%)
Platelet counts (10^9^/L)	100~300	≤234.00	37 (61.7%)	≤234.00	37 (61.7%)
		>234.00	23 (38.3%)	>234.00	23 (38.3%)
NLR	–	≤1.79	9 (15.0%)	≤1.79	9 (15.0%)
		>1.79	51 (85.0%)	>1.79	51 (85.0%)
PLR	–	≤243.75	52 (86.7%)	≤231.08	49 (81.7%)
		>243.75	8 (13.3%)	>231.08	11 (18.3%)
LMR	–	>6.24	10 (16.7%)	>6.24	10 (16.7%)
		≤6.24	50 (83.3%)	≤6.24	50 (83.3%)
SII	–	≤1,016.12	48 (80.0%)	≤1,016.12	48 (80.0%)
		>1,016.12	12 (20.0%)	>1,016.12	12 (20.0%)
TBIL (mg/dL)	0.29~1.64	≤0.74	47 (78.3%)	≤0.74	47 (78.3%)
		>0.74	13 (21.7%)	>0.74	13 (21.7%)
Albumin (g/L)	40~55	≤43.9	49 (81.7%)	≤45.8	54 (90.0%)
		>43.9	11 (18.3%)	>45.8	6 (10.0%)
PNI	–	≤47.65	52 (86.7%)	≤47.65	52 (86.7%)
		>47.65	8 (13.3%)	>47.65	8 (13.3%)

*PFS, progression-free survival; OS, overall survival; NLR, neutrophil-lymphocyte ratio; PLR, platelet-lymphocyte ratio; LMR, lymphocyte-monocyte ratio; SII, systemic immune-inflammation index; TBIL, total bilirubin; PNI, nutritional index*.

**Table 3:** Univariate and multivariate analysis of PFS.

**Variable**	**Parameter**	**Univariate analysis**		**Multivariate analysis**	
		**HR (95%CI)**	***P* value**	**HR (95% CI)**	***P* value**
Overall	–	–	–	–	
Sex	Female	1	0.379	–	
	Male	1.384 (0.671–2.855)			
Age (years old)	<60	1	0.937	–	
	≥60	1.067 (0.525–2.167)			
ECOG-PS	0–1	1	0.351	–	
	>1	1.446 (0.666–3.143)			
Area of brain involvement	Multiple	1	0.810	–	
	Single	1.091 (0.535–2.228)			
Deep structure involvement	Yes	1	0.249	–	
	No	1.531 (0.741–3.163)			
Surgical resection	No	1	0.284	–	
	Yes	1.775 (0.621–5.077)			
Rituximab combined treatment	No	1	0.897	–	
	Yes	1.053 (0.483–2.296)			
Consolidation treatment	Yes	1	0.375	–	
	No	1.754 (0.506–6.079)			
LDH	≤ULN	1	0.915	–	
	>ULN	1.050 (0.430–2.563)			
CSF protein	>45 mg/dL	1	0.844	–	
	≤45 mg/dL	1.123 (0.354–3.568)			
MSKCC	Class 1–2	1	0.096	–	
	Class 3	1.830 (0.899–3.723)			
Hemoglobin (g/dL)	>11.1	1	0.050^*^	3.940 (1.333–11.646)	0.013^*^
	≤11.1	2.240 (0.999–5.024)			
Neutrophil counts (10^9^/L)	≤3.28	1	0.013^*^	–	
	>3.28	3.379 (1.288–8.867)			
Lymphocyte counts (10^9^/L)	≤2.56	1	0.512	–	
	>2.56	1.421 (0.497–4.066)			
Monocyte counts (10^9^/L)	≤0.19	1	0.285	–	
	>0.19	2.194 (0.520–9.254)			
Platelet counts (10^9^/L)	≤234.00	1	0.015^*^	–	
	>234.00	2.407 (1.185–4.889)			
NLR	≤1.79	1	0.034^*^	10.548 (1.193–93.284)	0.034^*^
	>1.79	8.666 (1.176–63.850)			
PLR	≤243.75	1	0.017^*^	–	
	>243.75	2.807 (1.203–6.551)			
LMR	>6.24	1	0.169	–	
	≤6.24	2.311 (0.701–7.615)			
SII	≤1,016.12	1	0.000^*^	–	
	>1,016.12	4.069 (1.863–8.886)			
TBIL (mg/dL)	≤0.74	1	0.007^*^	3.429 (1.469–8.006)	0.004^*^
	>0.74	3.015 (1.353–6.717)			
Albumin (g/L)	≤43.9	1	0.078	–	
	>43.9	2.072 (0.921–4.665)			
PNI	≤47.65	1	0.017^*^	–	
	>47.65	2.807 (1.203–6.551)			

*PFS, progression-free survival; OS, overall survival; CI, confidence interval; HR, hazard ratio; ECOG-PS, Eastern Cooperative Oncology Group performance status; NR, not reached; LDH, lactate dehydrogenase; ULN, upper limit of normal; CSF, cerebrospinal fluid; MSKCC, Memorial Sloan Kettering Cancer Center; NLR, neutrophil-lymphocyte ratio; PLR, platelet-lymphocyte ratio; LMR, lymphocyte-monocyte ratio; SII, systemic immune-inflammation index; TBIL, total bilirubin; PNI, nutritional index*.

* *Statistically significant (p < 0.05)*.

Factors associated with worse OS in the univariate analysis were class 3 of MSKCC score (*p* = 0.033), neutrophil count (10^9^/L) > 3.28 (*p* = 0.035), lymphocyte counts (10^9^/L) ≤ 1.68 (*p* = 0.027), PLR > 231.08 (*p* = 0.039), lymphocyte–monocyte ratio (LMR) ≤ 6.24 (*p* = 0.048), SII > 1,016.12 (*p* = 0.000), TBIL (mg/dl) > 0.74 (*p* = 0.000), and PNI > 47.65 (*p* = 0.014) (Table 4). The Kaplan–Meier survival curves are shown in Figures 1B,D. LMR ≤ 6.24 (*p* = 0.039), SII > 1,016.12 (*p* = 0.012), and TBIL (mg/dl) > 0.74 (*p* = 0.009) were independently associated with worse OS in the multivariate analysis (Table 4).

**Table 4:** Univariate and multivariate analysis of OS.

**Variable**	**Parameter**	**Univariate analysis**		**Multivariate analysis**	
		**HR (95%CI)**	***P*-value**	**HR (95% CI)**	***P*-value**
0verall	–	–	–	–	
Sex	Female	1	0.734	–	
	Male	1.150 (0.514–2.570)			
Age (years old)	<60	1	0.723	–	
	≥60	1.153 (0.524–2.538)			
ECOG-PS	0–1	1	0.125	–	
	>1	2.158 (0.808–5.760)			
Area of brain involvement	Multiple	1	0.364	–	
	Single	1.439 (0.656–3.160)			
Deep structure involvement	No	1	0.731	–	
	Yes	0.870 (0.394–1.923)			
Surgical resection	No	1	0.649	–	
	Yes	1.281 (0.441–3.727)			
Rituximab combined treatment	No	1	0.400	–	
	Yes	1.505 (0.581–3.900)			
Consolidation treatment	Yes	1	0.819	–	
	No	1.185 (0.278–5.049)			
LDH	≤ULN	1	0.272	–	
	>ULN	1.682 (0.665–4.253)			
CSF protein	>45 mg/dL	1	0.686	–	
	≤45 mg/dL	1.271 (0.402–4.021)			
MSKCC	Class 1–2	1	0.033^*^	–	
	Class 3	2.402 (1.075–5.366)			
Hemoglobin (g/dL)	>15.0	1	0.258	–	
	≤15.0	24.260 (0.097–6,093.989)			
Neutrophil counts (10^9^/L)	≤3.28	1	0.035^*^	–	
	>3.28	3.185 (1.087–9.331)			
Lymphocyte counts (10^9^/L)	>1.68	1	0.027^*^	–	
	≤1.68	2.691 (1.121–6.461)			
Monocyte counts (10^9^/L)	≤0.19	1	0.143	–	
	>0.19	4.554 (0.599–34.609)			
Platelet counts (10^9^/L)	≤234.00	1	0.237	–	
	>234.00	1.613 (0.731–3.560)			
NLR	≤1.79	1	0.102	–	
	>1.79	31.334 (0.505–1,942.755)			
PLR	≤231.08	1	0.039^*^	–	
	>231.08	2.534 (1.047–6.135)			
LMR	>6.24	1	0.048^*^	24.040 (1.692–341.562)	0.019^*^
	≤6.24	7.555 (1.013–56.325)			
SII	≤1,016.12	1	0.000^*^	11.174 (2.490–50.147)	0.002^*^
	>1,016.12	4.573 (1.980–10.562)			
TBIL (mg/dL)	≤0.74	1	0.000^*^	5.245 (1.824–15.081)	0.002^*^
	>0.74	4.881 (2.029–11.741)			
Albumin (g/L)	≤45.8	1	0.084	–	
	>45.8	3.018 (0.860–10.589)			
PNI	≤47.65	1	0.014^*^	–	
	>47.65	3.227 (1.274–8.174)			

*OS, overall survival; CI, confidence interval; HR, hazard ratio; ECOG-PS, Eastern Cooperative Oncology Group performance status; NR, not reached; LDH, lactate dehydrogenase; ULN, upper limit of normal; CSF, cerebrospinal fluid; MSKCC, Memorial Sloan Kettering Cancer Center; NLR, neutrophil-lymphocyte ratio; PLR, platelet-lymphocyte ratio; LMR, lymphocyte-monocyte ratio; SII, systemic immune-inflammation index; TBIL, total bilirubin; PNI, nutritional index*.

* *Statistically significant (*p* < 0.05)*.

### Improvement of MSKCC Score and *C*-Index

**Figure 2:**
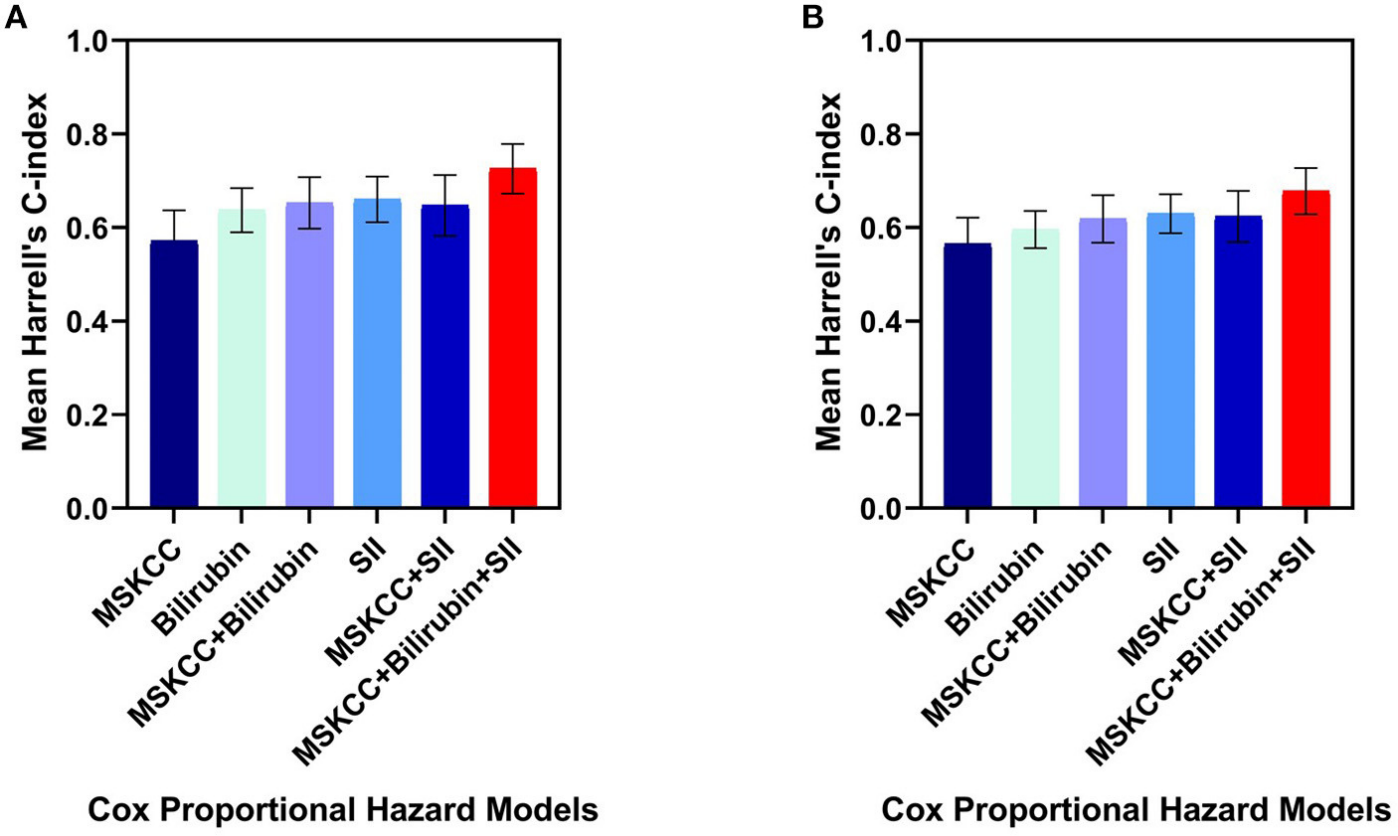
Harrell's *C*-index of five different Cox proportional hazard models on overall survival **(A)** and progression-free survival **(B)**: MSKCC score alone, bilirubin alone, SII alone, bilirubin plus MSKCC score, SII plus MSKCC score, bilirubin and SII plus MSKCC score separately. The error bars represent the standarad deviation of the Harrell's *C*-index. C-index, concordance index; SII, systemic immune-inflammation index; MSKCC, Memorial Sloan Kettering Cancer Center.

Of the three independent prognostic factors for OS in the multivariate analysis, only SII and TBIL achieved a *C*-index more than 0.6: 0.66 (SD 0.05) for SII and 0.63 (SD 0.05) for TBIL. Interestingly, when including SII, TBIL, and MSKCC in the multivariate Cox model, the *C*-index reached a value of 0.72 (SD 0.05) (Figure 2A). The prediction of PFS was in accordance with the OS presented in Figure 2B. Therefore, the combination of SII, TBIL, and MSKCC showed a stronger predictive ability than using the blood marker or MSKCC score separately.

## Discussion

The outcome prediction of PCNSL is still a challenge, and the prognostic models of PCNSL need to be validated and further improved based on the standard HD-MTX therapy. Our study retrospectively analyzed the prognostic role of pretreatment blood parameters in 60 PCNSL patients who received initial standard HD-MTX-based chemotherapy in a Chinese population cohort. In the multivariate analysis, we demonstrated that Hb, NLR, and TBIL had an independent prognostic value for PFS, while LMR, SII, and TBIL were independent prognostic factors for OS. Furthermore, better predictive ability for survival can be achieved by adding SII and TBIL to the MSKCC score.

The negative prognostic role of a low Hb level has been widely investigated in the solid and hematologic malignancies including PCNSL (14, 25, 26). Cytokines including interleukin-1 (IL-1), interleukin-6 (IL-6), and interleukin-10 (IL-10) have been demonstrated to inhibit the function of erythropoietin *in vivo*, and high IL-6 levels were thought to be the major factor for the development of anemia in DLBCL (27, 28); likewise, an elevated CSF level of IL-10 in PCNSL was associated with a shorter PFS (29). Moreover, previous studies found that NF-κb1-94ATTG deletion was associated with increased IL-6 and IL-10 in DLBCL (30), and IL-6 could induce the synthesis of hepcidin, which results in the insufficient supply of iron to erythropoiesis (27). Taken together, we evaluated that cytokine alternations in PCNSL may play a role for anemia. In parallel with a PCNSL study based on European populations which found that pretreatment anemia **(**defined as <12 g/dl in women and <13 g/dl in men) was robustly associated with poor prognosis (26), our study had a different cutoff value of ≤11.1 g/dl in all patients and found that patients with a low Hb level had an inferior outcome in PFS. But the cutoff for OS was 15 g/dl, which achieved maximum log-rank statistic and was not statistically significant in univariate analysis. Therefore, our study suggested a cutoff of ≤11.1 g/dl when applied in clinical practice.

There is increasing evidence that cancer-related inflammation (CRI) can promote malignant cell proliferation, invasion, and metastasis (31, 32). Tumor-associated macrophages (TAMs) play a leading role in CRI, whose prognostic value has been demonstrated in many lymphoproliferative malignancies (33). The prognostic value of other systemic inflammation response indicators represented by NLR and LMR has also been confirmed in several cancers (14, 17, 34–36). Neutrophils, as part of the innate immune system, can promote oncogenesis and suppress the function of lymphocytes which work in antitumor immunity (37). Monocytes, which could be recruited by large B-cell lymphoma cells via leukocyte attractant CCL5, can promote tumor cell survival and proliferation (38). It is consistent with our findings that NLR was an independent prognostic factor for disease progression and that a low LMR was significantly associated with worse OS. In the *in vitro* experiment, platelets activate tumor cell invasiveness by enhancing metalloproteinase-9 (MMP-9) secretion (39). However, platelet counts and PLR only correlated with worse survival in univariate analysis in our study.

When neutrophil, lymphocyte, and platelet counts are combined, SII has shown that its prognostic ability was superior to that of NLR, LMR, and PLR in lung cancer (40) and classical Hodgkin lymphoma (41). For the first time, we determined that SII was prognostic for both PFS and OS in univariate analysis and highly significant for PFS in multivariate analysis but lost its independent impact for OS in PCNSL. PNI is an indicator of systemic inflammation and nutritional status, but our study found it was associated with survival only in univariate analysis. Prospective and larger population studies are needed to further validate the prognostic value of these systemic inflammation response indicators in PCNSL.

The cutoff value of TBIL was 0.74 mg/dl in our study, and it was concluded that relatively elevated TBIL was a strong prognostic factor for both OS and PFS. The negative prognostic role of higher bilirubin levels (>0.52 mg/dl) has been identified in 515 patients with DLBCL in a previous study (14). The reason that bilirubin level influences outcomes in PCNSL remains speculative, as the cutoff values of these two studies were all in the normal range of the average population. There are two related findings that ought to be considered. First, HD-MTX treatment was proven to be hepatically toxic and can lead to a decreased reserve capacity of the liver (42). Second, the variability of the UDP-glucuronosyltransferase 1A (UGT1A) family of genes was identified as a genetic risk factor in a variety of cancers (43). Thus, it is possible that the variability of UGT1A in lymphomas may result in a different catalytic efficacy of the glucuronidation system, determining diverse levels of bilirubin and outcomes of HD-MTX treatment in PCNSL. Furthermore, we tested the prognostic role of a higher TBIL level, 1 mg/dl; this cutoff value was not statistically significant in univariate analysis. The highest TBIL level was 1.47 mg/dl in our research cohort, demonstrating that a cutoff value within the normal range of TBIL was prognostic. It is hard to apply this index into clinical practice since it is within normal laboratory values, but our work may encourage more verification and exploration into this setting. Additionally, more attention could be given to the change of liver function parameters during HD-MTX treatment, and more liver-protecting treatments might be taken.

Our results indicated that the MSKCC score when combined with SII and TBIL had a better prognostic value for PCNSL patients receiving HD-MTX-based chemotherapy. SII and TBIL might be effective supplementary factors to improve the predictive ability of previous prognostic models. Since the role of the MSKCC score is still controversial, we verified its role in patients treated with standard HD-MTX-based treatment and improved it using simple and convenient parameters in clinical practice. The choices of induction chemotherapy and consolidation treatment are diverse in PCNSL; thus, a better disease risk stratification score can help with clinical decision making and develop risk-adapted treatments.

The impact of surgical excision on survival in PCNSL has not been firmly validated (44, 45). Our cohort had a relatively high proportion (78.3%) of patients who received cytoreductive surgery, but no survival benefit was shown. The addition of rituximab as another important therapy for PCNSL remains controversial (46–48), and no significant difference was found in our study. A previous study suggested that ASCT was better than WBRT in consolidation treatment (49), which was not confirmed in our study, possibly because only two patients have been treated with ASCT and one died of pneumonia after ASCT. Thus, further study is required to determine better therapeutic options in PCNSL.

Nevertheless, our study had several limitations. First, this study was retrospective and monocentric; therefore, selection and information bias could not have been avoided. Besides, 17 (28.3%) patients received glucocorticoid treatment before diagnosis which could have influenced the blood test, in particular, the neutrophil counts. Second, our study cohorts were small and based on Chinese populations, which limited its validation of previous models developed mainly on Western populations. Despite these limitations, we studied the prognostic value of LMR, SII, and PNI for the first time in PCNSL who received initial standard HD-MTX-based chemotherapy and improved the MSKCC score.

## Conclusion

In this study, we show that NLR, LMR, Hb, SII, and TBIL are simple prognostic factors in PCNSL. SII and TBIL are effective and promising blood markers to improve the predictive ability of the MSKCC score. Further studies are needed to verify these markers and the improved MSKCC score.

## Data Availability Statement

The raw data supporting the conclusions of this article will be made available by the authors on reasonable request, without undue reservation.

## Ethics Statement

The studies involving human participants were reviewed and approved by Ethics Administration Office of West China Hospital, Sichuan University. Written informed consent for participation was not required for this study in accordance with the national legislation and the institutional requirements.

## Author Contributions

QL, CY, YW, and LZ: conception and design. YW and LZ: administrative support. QL, CF, WW, and CY: collection and analysis of data. All authors: manuscript writing, final approval of manuscript, and accountable for work.

## Conflict of Interest

The authors declare that the research was conducted in the absence of any commercial or financial relationships that could be construed as a potential conflict of interest.
